# *Clonorchis sinensis* infection amplifies hepatocellular carcinoma stemness, predicting unfavorable prognosis

**DOI:** 10.1371/journal.pntd.0011906

**Published:** 2024-01-29

**Authors:** Qiumei Lin, Zeli Tang, Yuling Qin, Xueling Deng, Caibiao Wei, Fengfei Liu, Xiaolan Pan, Dengyu Liu, Tingzheng Zhan, Min Fang

**Affiliations:** 1 Department of Clinical Laboratory, Guangxi Medical University Cancer Hospital, Nanning, People’s Republic of China; 2 Department of Cell Biology and Genetics, School of Basic Medical Sciences, Guangxi Medical University, Nanning, People’s Republic of China; 3 Department of Parasitology, School of Basic Medical Sciences, Guangxi Medical University, Nanning, People’s Republic of China; 4 Engineering Research Center for Tissue & Organ Injury and Repair Medicine, Guangxi Medical University Cancer Hospital, Nanning, People’s Republic of China; James Cook University Division of Tropical Health and Medicine, AUSTRALIA

## Abstract

**Background:**

Extensive evidence links *Clonorchis sinensis* (*C*. *sinensis*) to cholangiocarcinoma; however, its association with hepatocellular carcinoma (HCC) is less acknowledged, and the underlying mechanism remains unclear. This study was designed to investigate the association between *C*. *sinensis* infection and HCC and reveal the relationship between *C*. *sinensis* infection and cancer stemness.

**Methods:**

A comprehensive analysis of 839 HCC patients categorized into *C*. *sinensis* (-) HCC and *C*. *sinensis* (+) HCC groups was conducted. Chi-square and Mann–Whitney U tests were used to assess the association between *C*. *sinensis* infection and clinical factors. Kaplan–Meier and Cox regression analyses were used to evaluate survival outcomes. Immunohistochemistry was used to determine CK19 and EpCAM expression in HCC specimens.

**Results:**

Compared to *C*. *sinensis* (-) HCC patients, *C*. *sinensis* (+) HCC patients exhibited advanced Barcelona Clinic Liver Cancer (BCLC) stage, higher male prevalence and more liver cirrhosis as well as elevated alpha-fetoprotein (AFP), carbohydrate antigen 19–9 (CA19-9), eosinophil, complement 3 (C3), and complement 4 (C4) values. *C*. *sinensis* infection correlated with shorter overall survival (OS) (*p* < 0.05) and recurrence-free survival (RFS) (*p* < 0.05). Furthermore, Cox multivariate analysis revealed that *C*. *sinensis* infection was an independent prognostic factor for OS in HCC patients. Importantly, *C*. *sinensis* infection upregulated the expression of HCC cancer stem cell markers CK19 and EpCAM.

**Conclusion:**

HCC patients with *C*. *sinensis* infection exhibit a poor prognosis following hepatectomy. Moreover, *C*. *sinensis* infection promotes the acquisition of cancer stem cell-like characteristics, consequently accelerating the malignant progression of HCC.

**Author summary:**

*Clonorchis sinensis* (*C*. *sinensis*) is a prominent food-borne parasite prevalent in regions such as China, particularly in Guangxi. *C*. *sinensis* has been associated with various hepatobiliary system injuries, encompassing inflammation, periductal fibrosis, cholangiocarcinoma and even hepatocellular carcinoma (HCC). A substantial body of evidence links *C*. *sinensis* to cholangiocarcinoma, However, the connection between *C*. *sinensis* and HCC and the intricate mechanisms underlying its contribution to HCC development remain incompletely elucidated. Our study demonstrates clear clinicopathological associations between *C*. *sinensis* and HCC, such as gender, BCLC stage, liver cirrhosis, MVI, AFP, CA19-9, circulating eosinophils and complements. Furthermore, we found that the co-occurrence of *C*. *sinensis* exhibited a significant association with shorter OS and RFS in patients diagnosed with HCC. A major finding was that *C*. *sinensis* infection promotes the acquisition of cancer stem cell-like characteristics, consequently accelerating the malignant progression of HCC. Our results provide a more comprehensive comprehension of the interplay between *C*. *sinensis* and HCC, shedding fresh light on the carcinogenic potential of *C*. *sinensis*.

## Introduction

Hepatocellular carcinoma (HCC) remains an important global health burden, contributing substantially to cancer-related mortality worldwide [1,2]. Notably, China shoulders the highest burden of HCC in the world. In 2019, approximately 300,000 people in China were diagnosed with HCC, which tragically resulted in nearly 200,000 HCC-related deaths [3]. Despite recent advancements, the prognosis for HCC patients remains grim, with an overall 5-year survival rate of 25–30% and recurrence/metastasis rates of 50–70% within 5 years following radical resection [3]. Chronic viral hepatitis, alcohol-associated cirrhosis, and nonalcoholic fatty liver disease have been proven to be the main risk factors for developing HCC [1,4–8]. Mounting evidence suggests that certain parasitic infections, such as *Clonorchis sinensis* (*C*. *sinensis*), may play a role in the development and prognosis of HCC [9–11].

*C*. *sinensis*, recognized as one of the most severe food-borne parasites, predominantly infects the biliary system of humans and other mammals [12]. Its prevalence is notably high in East Asian regions, including China, Korea, and Vietnam, presenting an important public health concern [13,14]. Notably, China bears the greatest burden of *C*. *sinensis* infection, with an estimated 35 million people infected worldwide, of which approximately 15 million cases are in China [14,15]. The presence of *C*. *sinensis* in the bile ducts can lead to biliary inflammation, biliary obstruction, liver cirrhosis and even carcinogenesis [10,16–18]. Furthermore, epidemiological and clinical studies have unveiled disconcerting links between *C*. *sinensis* infection and the prognosis of HCC patients, suggesting that it may serve as a substantial risk factor for HCC [10]. The carcinogenesis of *C*. *sinensis* infection involves various factors, such as mechanical obstruction and damage to the biliary barrier system, consisting of infection-related inflammation, pathological effects from excretory-secretory products (ESPs), enhanced features of epithelial–mesenchymal transition, and immunopathology imbalance [10,19–21]. However, the intricate mechanisms underlying *C*. *sinensis*’s contribution to HCC development remain incompletely elucidated.

Cancer stem cells (CSCs) account for a small subset of tumor cells with long-term tumorigenic capacity and play a pivotal role in cancer development and therapy resistance [22]. Emerging evidence has underscored that the stemness of liver cancer cells is a pivotal contributor to the recurrence and metastasis of HCC [23]. Tumors that harbor an abundant CSC population may signal a poor clinical outcome in patients with HCC [24]. The CSC biomarkers of HCC include epithelial cell adhesion molecule (EpCAM), CK19, CD44, CD133, CD24, and CD13 [24]. Further investigations are needed to explore whether *C*. *sinensis* promotes the stemness of liver cancer cells.

Hence, systematic endeavors have been undertaken to scrutinize the correlation between *C*. *sinensis* infection and clinical parameters in HCC, aiming to explore the predictive significance of *C*. *sinensis* infection in patients with HCC following hepatectomy. Furthermore, this article specifically focuses on the involvement of *C*. *sinensis* in liver cancer stemness, with the goal of uncovering the underlying mechanisms responsible for the unfavorable prognosis observed in HCC patients concomitantly afflicted by *C*. *sinensis*.

## Patients and methods

### Ethics statement

This research was approved by the Medical Ethics Committee of Guangxi Medical University Cancer Hospital (LW2023135) and conducted following the ethical principles outlined in the Helsinki Declaration of 1964 and its subsequent amendments or other ethical standards with equivalent requirements. To ensure patient confidentiality, the identities of the individuals included in this study were anonymized using computer-generated ID numbers. On admission, all patients provided written consent for their anonymized medical data to be analyzed and published for research purposes.

### Study population and data collection

This study was conducted at Guangxi Medical University Cancer Hospital from October 2013 to December 2021. This study conformed to the ethics of the Declaration of Helsinki and was approved by the Ethics Committee of Guangxi Medical University Cancer Hospital. A total of 1,908 HCC patients without cardiovascular diseases (including hypertension and coronary heart disease), chronic respiratory diseases and diabetes mellitus underwent radical resection. The inclusion criteria were as follows: (1) histologically confirmed diagnosis of HCC through postoperative pathological analysis; (2) the absence of prior anticancer treatment; (3) the absence of concurrent malignancies; (4) availability of comprehensive laboratory, pathological, and follow-up data. Exclusion criteria, on the other hand, included the following: (1) previous antitumor treatments such as transcatheter arterial chemoembolization, chemotherapy, or radiofrequency ablation (N = 187); (2) presence of malignancies other than HCC (N = 72); (3) cases of recurrent HCC (N = 143); and (4) unavailability of comprehensive laboratory, pathological, and follow-up data (N = 667).

Clonorchiasis is caused by infection with *C*. *sinensis* [25], and the diagnostic criteria for clonorchiasis are as follows, with any one of the subsequent conditions deemed sufficient for establishing a diagnosis [9,12,18,26,27]: (1) preoperative imaging (MRI, CT, microscopy or ultrasound) confirming the presence of *C*. *sinensis* eggs or adult worms on the intrahepatic bile ducts; (2) a preoperative positive result in serologic ELISA testing; (3) intraoperative or postoperative pathological examination revealing the presence of adult *C*. *sinensis* in the liver or gallbladder; (4) preoperative fecal examination showing the presence of *C*. *sinensis* eggs. Finally, according to whether HCC patients were infected with *C*. *sinensis*, we assigned them to a *C*. *sinensis* (+) HCC group (*C*. *sinensis* positive) or a *C*. *sinensis* (-) HCC group (*C*. *sinensis* negative). Consequently, the clinicopathological data related to HCC following hepatectomy retrospectively analyzed in this study comprised that of 839 patients, among whom 87 exhibited concomitant *C*. *sinensis* infection along with HCC.

Data collection was performed by two independent investigators, QML and ZLT, with validation by a third investigator, YLQ. The data collection process encompassed multiple aspects, including the following: (1) general information: gender and age; (2) hematological tests including various tumor markers (alpha-fetoprotein (AFP), cancer antigen 19–9 (CA19-9), carcinoembryonic antigen (CEA)), hepatitis B surface antigen(HBsAg), liver function-related indicators (total bilirubin (TBil), aspartate transaminase (AST), and alanine transaminase (ALT)), complete blood count (white blood cells (WBC), platelets (PLT), neutrophils (NEU), eosinophils (EOS), lymphocytes (LYM)), circulating immunity indicators (percentages of CD4 T lymphocytes, CD8 T lymphocytes, B lymphocytes, and NK cells, circulating immunoglobulins (IgG, IgM and IgA) and circulating complement (C3 and C4)); (3) pathological indicators liver cirrhosis, node number, tumor size, tumor differentiation degree (the criteria of the Edmondson-Steiner histological grading system are as follows: grade I, well-differentiated; grade II, moderately differentiated; grade III-IV, poorly differentiated) and the presence of microvascular invasion (MVI) [28].

### Laboratory methods

The concentrations of TBiL, ALT, AST, IgG, IgM, IgA, C3 and C4 were determined utilizing a Siemens ADVIA 2400 chemistry analyzer. Hematological parameters were assessed through a Mindray Coulter CAL8000 blood analyzer. Meanwhile, the levels of AFP, CA19-9, and CEA were quantified by employing an Abbott I2000SR analyzer. The proportions of circulating CD4 T lymphocytes, CD8 T lymphocytes, B lymphocytes, and NK cells were analyzed utilizing BD flow cytometry.

### Follow-up routine

Patient follow-up was diligently managed by professional staff, utilizing a telephone contact or outpatient monitoring systems to ascertain patient disease status or date of mortality. The identification of tumor recurrence hinged on an analysis of radiological observations extracted from CT or MRI scans, with a particular emphasis on discerning characteristic enhancement patterns indicative of intrahepatic recurrence. When dealing with extrahepatic tumors or those displaying atypical HCC imaging traits, verification was secured through biopsy. Patients underwent systematic monitoring at designated time intervals after their surgical procedures. The calculation of overall survival (OS) entailed determining the span between the date of hepatectomy and the date of death or the last follow-up, with September 30, 2022, as the end date. Recurrence-free survival (RFS) was defined as the temporal gap between the date of hepatectomy and the occurrence of HCC recurrence or the last follow-up, again until September 30, 2022.

### Histology and immunohistochemistry

Paraffin-embedded tissue samples from HCC patients were skillfully sectioned into slices measuring 4 μm in thickness. These sections underwent deparaffinization using xylene and were subsequently rehydrated through a series of alcohol washes. To ensure optimal quality, all sections underwent microwave-based heating for repair, while endogenous peroxidase activity was quenched using a 3% H_2_O_2_ solution. Following these preparatory steps, the sections were subjected to an overnight incubation at a temperature of 4°C utilizing anti-CK19 antibody (dilution 1:150; Maixim, Kit-0030) or anti-EpCAM antibody (dilution 1:500; Thermo Fisher, Clone: 1B7). The subsequent immunohistochemical analysis was executed using the DAKO EnVision detection system.

To ensure unbiased evaluation, two independent pathologists, who remained unaware of the associated clinical data, meticulously assessed the immunohistochemical staining scores for CK19 and EpCAM within the liver tissues by lab standard operation files. This evaluation was conducted through a semi-quantitative approach. This evaluation was conducted through a semiquantitative approach. The staining scores were categorized into four distinct levels as follows: 0 (negative), 1 (weak), 2 (moderate), and 3 (strong). Specifically, high expression was defined as a staining score exceeding 2, wherein a minimum of 75% of malignant cells exhibited positive staining. In contrast, moderate expression referred to a staining score of 2, accompanied by at least 25% of malignant cells showing positive staining. Last, low expression entailed a staining score below 2, denoting that less than 25% of malignant cells exhibited positive staining.

### Statistical analysis

Statistical analysis was conducted using IBM SPSS Statistics software version 26.0 and R version 4.2.1. Intergroup differences for categorical data presented as ratios were compared using either the Chi-square test or Fisher’s exact test. The Mann–Whitney U test was used to compare nonnormal continuous data. Survival curves were generated using the Kaplan–Meier method, and intergroup comparisons of OS and RFS rates were performed using the log-rank test. Univariate and multivariate prognostic analyses were conducted using the Cox regression model. In the multivariate Cox analysis, variables that demonstrated statistically significant differences (*p* < 0.05) in the univariate analysis were included to identify independent risk factors for OS and RFS. All statistical tests were two-sided, and *p* < 0.05 was considered statistically significant.

## Results

### Population characteristics

**Fig 1:**
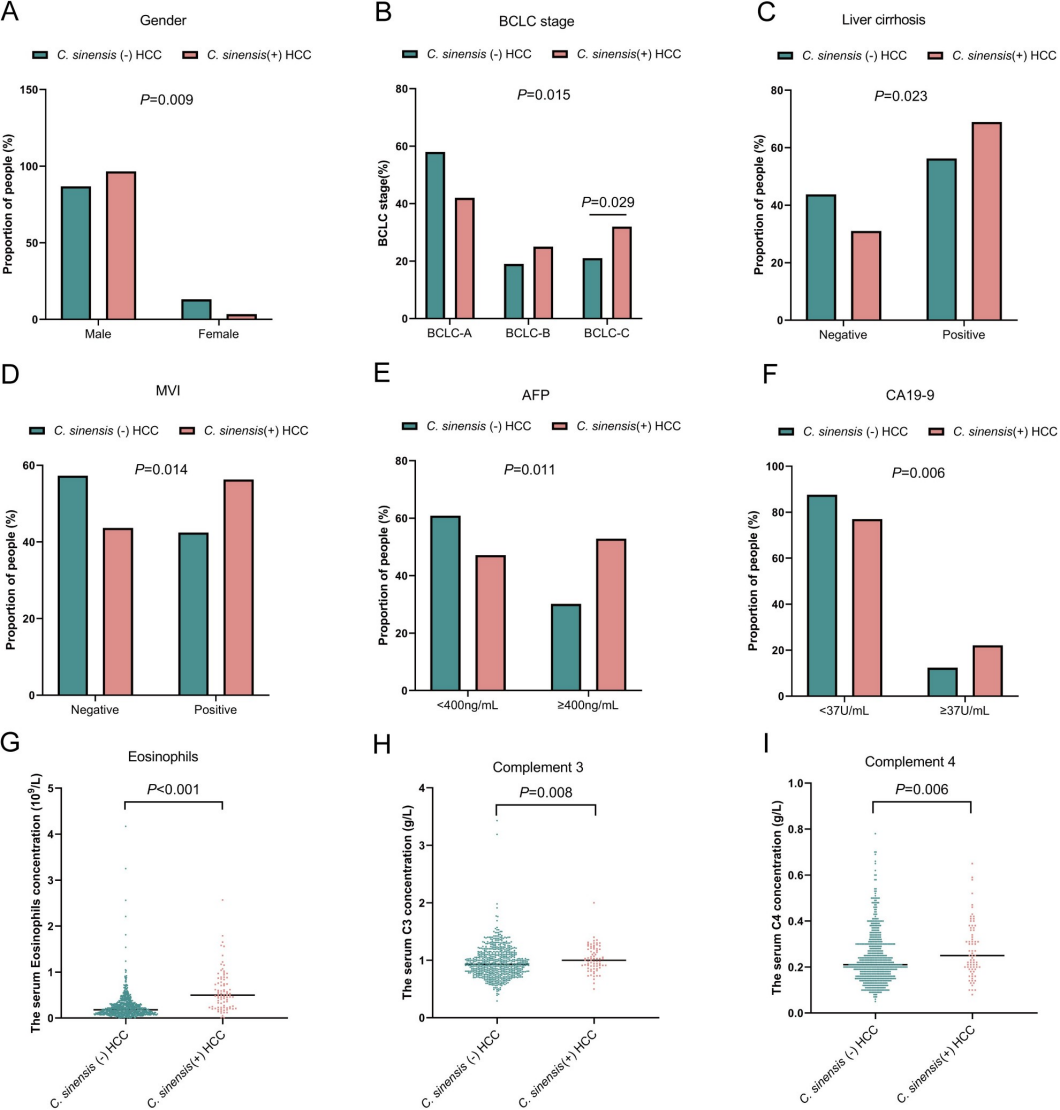
Comparative analysis of clinical characteristics and continuous variables in the *C*. ***sinensis* (+) and *C*. *sinensis* (-) HCC groups.** (A) Percentage distribution of gender; (B) Barcelona Clinic Liver Cancer (BCLC) stage; (C) presence of liver cirrhosis; (D) presence of microvascular invasion (MVI); (E) alpha-fetoprotein (AFP) levels; and (F) carbohydrate antigen 19–9 (CA19-9) levels in patients with hepatocellular carcinoma (HCC) stratified by *Clonorchis sinensis* (*C*. *sinensis*) infection status; statistical significance was assessed using the Chi-square test; (G) scatter diagram illustrating the distribution of eosinophils, (H) complement 3 (C3), and (I) complement 4 (C4) levels in patients with *C*. *sinensis* (+) HCC and *C*. *sinensis* (-) HCC; the Mann–Whitney U test was employed for statistical analysis. **Abbreviations:** *C*. *sinensis*, Clonorchis sinensis; HCC, hepatocellular carcinoma; BCLC, Barcelona Clinic Liver Cancer; MVI, microvascular invasion; AFP, alpha-fetoprotein; CA19-9, carbohydrate antigen 199.

Table 1 provides an overview of the baseline characteristics of all the included patients. A total of 839 patients were enrolled in this study, including 786 males (87.84%), with a higher prevalence of males in the *C*. *sinensis* (+) HCC group compared to the *C*. *sinensis* (-) HCC group (86.8% vs. 96.6%, *p*<0.05). *C*. *sinensis* (+) HCC patients had a more advanced BCLC stage than *C*. *sinensis* (-) HCC patients (BCLC B-C 57.47% vs. 41.49%, *p*<0.05). Furthermore, *C*. *sinensis* (+) HCC patients had a higher prevalence of liver cirrhosis (68.97% vs. 56.25%) and MVI (56.32% vs. 42.44%) than *C*. *sinensis* (-) HCC patients (*p* <0.05 for all). *C*. *sinensis* (+) HCC patients had higher AFP, CA19-9, eosinophil, C3 and C4 values than *C*. *sinensis* (-) HCC patients (*p* <0.05 for all). However, no statistically significant differences were observed in terms of age, tumor size, number of tumors, Edmondson grade, CEA, HBsAg, HCV, ALB, ALT, AST, TBil, neutrophils, lymphocytes, circulating immune cells, or circulating immunoglobulins. Fig 1 visualizes the clinical features with significant differences in Table 1.

**Table 1:** Patient demographics and clinical characteristics.

Characteristics	*C*. *sinensis* (-) HCC	*C*. *sinensis* (+) HCC	
	No. (%)	No. (%)	*P-value*
Total	752	87	
Gender			0.009
Male	653(86.84%)	84(96.55%)	
Female	99(13.16%)	3(3.45%)	
Age			0.218
<60	541(71.94%)	68(78.16%)	
≥60	211(28.06%)	19(21.84%)	
BCLC stage			0.004
A	440(58.51%)	37(42.53%)	
B~C	312(41.49%)	50(57.47%)	
Tumor size			0.904
<5cm	332(44.15%)	39(44.83%)	
≥5cm	420(55.85%)	48(55.17%)	
Liver cirrhosis			0.023
Negative	329(43.75%)	27(31.03%)	
Positive	423(56.25%)	60(68.97%)	
Edmondson grade			0.870
I-II	370(49.20%)	42(48.28%)	
III-IV	382(50.80%)	45(51.72%)	
Number of tumors			0.754
<2	612(81.38%)	72(82.76%)	
≥2	140(18.62%)	15(17.24%)	
MVI			0.014
Negative	432(57.29%)	38(43.68%)	
Positive	320(42.44%)	49(56.32%)	
CEA			0.881
<5ng/mL	678(90.16%)	78(89.66%)	
≥5ng/mL	74(9.84%)	9(10.34%)	
AFP			0.011
<400ng/mL	458(60.90%)	41(47.13%)	
≥400ng/mL	294(39.10%)	46(52.87%)	
CA19-9			0.006
<37U/mL	659(87.63%)	67(77.01%)	
≥37U/mL	93(12.37%)	20(22.99%)	
HBsAg			0.068
Negative	123(16.36%)	21(24.14%)	
Positive	629(83.64%)	66(75.86%)	
Anti-HCV			1.000
Negative	744(98.9%)	86(100.00%)	
Positive	8(1.1%)	0(0.00%)	
ALB(g/L)	38.3(22–65.3)	38.8(30.6–56)	0.561
ALT(U/L)	34(0–562)	34(8–309)	0.857
AST(U/L)	39(11–407)	41(10.3–265)	0.108
TBil(μmol/mL)	13.7(2.4–452.5)	13.4(3.54–84.8)	0.611
Neutrophil(x10^9^/L)	3.475(0.86–14.16)	3.9(0.79–10.83)	0.116
Lymphocyte(x10^9^/L)	1.72(0.46–5.57)	1.8(0.73–3.03)	0.422
Eosinophils(x10^9^/L)	0.18(0–4.17)	0.495(0.01–1.79)	<0.001
CD3(%)	65.9(0.43–89.7)	65.735(46.53–93.6)	0.563
CD4(%)	38.65(12.5–67.7)	40(20.1–59.6)	0.320
CD8(%)	20.2(5.2–45.9)	18.95(8.5–38.6)	0.299
NK(%)	12(1.1–52.9)	12.45(4.87–33.9)	0.522
B(%)	13.3(1–32.2)	13.8(3.33–41.6)	0.446
IgG(g/L)	13.06(4.3–29.86)	12.74(6.21–28.19)	0.161
IgM(g/L)	1.09(0.12–19.06)	1.16(0.29–3.21)	0.697
IgA(g/L)	2.64(0.03–12.46)	2.71(1.08–5.67)	0.849
C3(g/L)	0.93(0.29–3.43)	1(0.5–2)	0.008
C4 (g/L)	0.21(0.05–0.78)	0.25(0.08–0.65)	0.006

Continuous variables are reported as median (range).

**Abbreviations:** *C*. *sinensis*, *Clonorchis sinensis*; HCC, hepatocellular carcinoma; BCLC, Barcelona Clinic Liver Cancer; MVI, microvascular invasion; CEA, carcinoembryonic antigen; AFP, alpha-fetoprotein; CA19-9, carbohydrate antigen 199; HBsAg, hepatitis B surface antigen; Anti-HCV, hepatitis C virus antibody; ALB, albumin; ALT, alanine aminotransferase; AST, aspartate aminotransferase; TBil, total bilirubin; CD3, CD3 T lymphocytes; CD4, CD4 T lymphocytes; CD8, CD8 T lymphocytes; NK, natural killer cells; B, B lymphocytes; IgG, immunoglobulin G antibodies; IgM, immunoglobulin M antibodies; IgA, immunoglobulin A antibodies; C3, complement 3; C4, complement 4.

### The association between *C*. *sinensis* infection and poor prognosis in HCC patients

**Fig 2:**
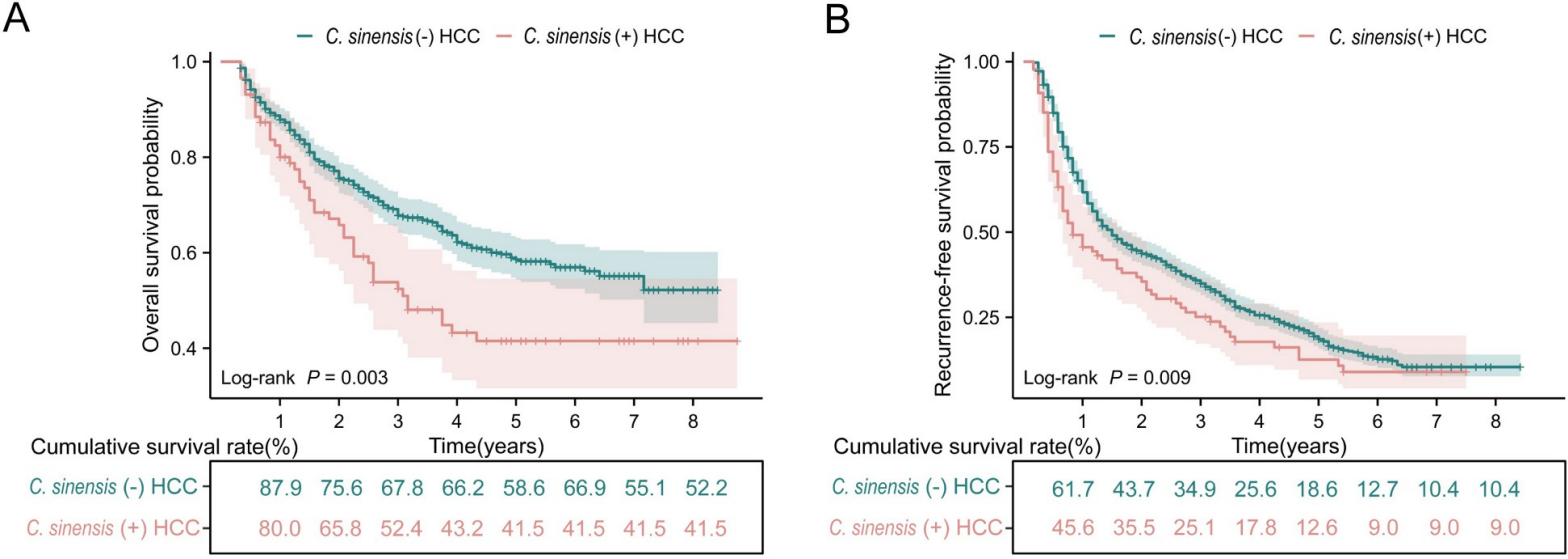
The influence of *C*. *sinensis* on the prognosis of HCC patients after liver resection. **(A)** *C*. *sinensis* for overall survival; **(B)** *C*. *sinensis* for recurrence-free survival. **Abbreviations:** *C*. *sinensis*, *Clonorchis sinensis*; HCC, hepatocellular carcinoma.

After a median follow-up of 30 months (range 4–105 months), the three- and five-year OS rates in the whole series were 66.1% and 56.6%, respectively. At the last follow-up, 548 (65.3%) patients were alive, including 144 (17.2%) without evidence of disease. In the *C*. *sinensis* (-) HCC group, a total of 274 (36.4%) cases of death were recorded, whereas in the *C*. *sinensis* (+) HCC group, 47 (54.0%) cases of death were documented. *C*. *sinensis* (+) HCC patients had worse OS than *C*. *sinensis* (-) HCC patients (at three years, 67.8% vs. 52.4%; at five years, 58.6% vs. 41.5%, *p*<0.05, Fig 2A). The enrolled patients had an RFS follow-up period of 2 to 101 months, with a median of 17 months. RFS rates were lower in *C*. *sinensis* (+) HCC patients than in *C*. *sinensis* (-) HCC patients (at three years, 34.9% vs. 25.1%; at five years; 18.6% vs. 12.6%, *p*<0.05, Fig 2B).

A total of 31 different factors were included in the multivariate Cox regression analysis for OS and RFS. Univariate analysis was used to analyze factors that could influence OS or RFS in HCC patients, including *C*. *sinensis*. In this study, 15 variables were identified as important predictive factors for OS, including *C*. *sinensis*. In the multivariate analysis (Tables 2 and 3), *C*. *sinensis* positivity was an independent prognostic risk factor for OS (hazard ratio = 1.528, confidence interval 95% 1.067–2.187, *p* < 0.05). However, *C*. *sinensis* was not an independent prognostic factor for RFS.

**Table 2:** Univariate and multivariate analysis of prognostic factors for overall survival (OS) in 839 HCC patients.

Characteristics	Univariate analysis		Multivariate analysis	
	HR (95% CI)	*P*	HR (95% CI)	*P*
*C*. *sinensis* (negative vs positive)	1.605(1.168–2.206)	0.004	1.528(1.067–2.187)	0.021
Gender (male vs female)	1.565(1.046–2.342)	0.029	0.998(0.638–1.560)	0.993
Age (<60 vs ≥60)	0.971(0.746–1.263)	0.824		
BCLC stage (A vs B~C)	2.816(2.219–3.575)	<0.001	1.738(1.278–2.363)	<0.001
Tumor size (<5cm vs ≥5cm)	1.970(1.538–2.522)	<0.001	1.292(0.946–1.766)	0.107
Liver cirrhosis (negative vs positive)	1.173(0.929–1.481)	0.181		
Edmondson grade (I-II vs III-IV)	1.774(1.400–2.248)	<0.001	1.395(1.069–1.822)	0.014
Number of tumors (<2 vs ≥2)	1.368(1.040–1.801)	0.025	1.166(0.838–1.622)	0.363
MVI (negative vs positive)	3.136(2.458–4.001)	<0.001	2.105(1.574–2.814)	<0.001
CEA (<5ng/mL vs ≥5ng/mL)	1.222(0.846–1.764)	0.285		
AFP (<400ng/mL vs ≥400ng/mL)	1.590(1.263–2.001)	<0.001	1.014(0.775–1.326)	0.922
CA19-9 (<37U/mL vs ≥37U/mL)	1.661(1.253–2.201)	<0.001	1.745(1.267–2.402)	0.001
ALB (<35g/L vs ≥35g/L)	0.787(0.590–1.050)	0.104		
ALT (<40U/L vs ≥40U/L)	1.110(0.878–1.403)	0.383		
AST (<40U/L vs ≥40U/L)	1.960(1.547–2.484)	<0.001	1.400(1.049–1.867)	0.022
TBil (<17.1μmol/mL vs ≥17.1μmol/mL)	1.008(0.785–1.295)	0.949		
HBsAg (negative vs positive)	1.040(0.765–1.413)	0.804		
Anti-HCV (negative vs positive)	0.706(0.176–2.837)	0.624		
Neutrophil (<3.52 x10^9^/L vs ≥3.52 x10^9^/L)	1.587(1.257–2.004)	<0.001	1.387(1.047–1.837)	0.023
Lymphocyte (<1.72x10^9^/L vs ≥1.72 x10^9^/L)	0.762(0.605–0.960)	0.021	0.798(0.613–1.040)	0.095
Eosinophils (<0.2x10^9^/L vs ≥0.2 x10^9^/L)	1.225(0.972–1.544)	0.085		
CD3 (<65.90% vs ≥65.90%)	0.826(0.639–1.068)	0.144		
CD4 (<38.80% vs ≥38.80%)	1.059(0.820–1.368)	0.662		
CD8 (<20.15% vs ≥20.15%)	0.853(0.660–1.102)	0.224		
NK (<12.10% vs ≥12.10%)	1.196(0.925–1.548)	0.173		
B (<13.40% vs ≥13.40%)	0.997(0.772–1.287)	0.979		
IgG (<12.995g/L vs ≥12.995g/L)	0.745(0.578–0.961)	0.023	0.659(0.507–0.857)	0.002
IgM (<1.1g/L vs ≥1.1g/L)	1.032(0.802–1.327)	0.809		
IgA (<2.645g/L vs ≥2.645g/L)	1.162(0.903–1.497)	0.244		
C3 (<0.94g/L vs ≥0.94g/L)	1.607(1.239–2.084)	<0.001	1.117(0.828–1.506)	0.469
C4 (<0.21g/L vs ≥0.21g/L)	1.509(1.162–1.960)	0.002	0.958(0.705–1.301)	0.783

**Abbreviations:** *C*. *sinensis*, *Clonorchis sinensis*; BCLC, Barcelona Clinic Liver Cancer; MVI, microvascular invasion; CEA, carcinoembryonic antigen; AFP, alpha-fetoprotein; CA19-9, carbohydrate antigen 199; HBsAg, hepatitis B surface antigen; Anti-HCV, hepatitis C virus antibody; ALB, albumin; ALT, alanine aminotransferase; AST, aspartate aminotransferase; TBil, total bilirubin; CD3, CD3 T lymphocytes; CD4, CD4 T lymphocytes; CD8, CD8 T lymphocytes; NK, natural killer cells; B, B lymphocytes; IgG, immunoglobulin G antibodies; IgM, immunoglobulin M antibodies; IgA, immunoglobulin A antibodies; C3, complement 3; C4, complement 4.

**Table 3:** Univariate and multivariate analysis of prognostic factors for recurrence-free survival (RFS) in 839 HCC patients.

Characteristics	Univariate analysis		Multivariate analysis	
	HR (95% CI)	*P*	HR (95% CI)	*P*
*C*. *sinensis* (negative vs positive)	1.372(1.076–1.750)	0.011	1.278(0.970–1.684)	0.081
Gender (male vs female)	1.340(1.051–1.709)	0.018	1.141(0.877–1.485)	0.326
Age (<60 vs ≥60)	1.086(0.919–1.285)	0.333		
BCLC stage (A vs B~C)	1.594(1.370–1.854)	<0.001	1.229(1.026–1.472)	0.025
Tumor size (<5cm vs ≥5cm)	1.262(1.083–1.470)	0.003	1.032(0.860–1.237)	0.736
Liver cirrhosis (negative vs positive)	1.275(1.093–1.487)	0.002	1.188(1.002–1.408)	0.047
Edmondson grade (I-II vs III-IV)	1.138(0.978–1.324)	0.093		
Number of tumors (<2 vs ≥2)	1.121(0.927–1.356)	0.239		
MVI (negative vs positive)	1.477(1.269–1.718)	<0.001	1.258(1.054–1.500)	0.011
CEA (<5ng/mL vs ≥5ng/mL)	1.120(0.873–1.437)	0.372		
AFP (<400ng/mL vs ≥400ng/mL)	1.276(1.095–1.487)	0.002	1.025(0.862–1.219)	0.777
CA19-9 (<37U/mL vs ≥37U/mL)	0.853(0.680–1.070)	0.169		
ALB (<35g/L vs ≥35g/L)	0.861(0.708–1.048)	0.135		
ALT (<40U/L vs ≥40U/L)	1.143(0.980–1.334)	0.088		
AST (<40U/L vs ≥40U/L)	1.460(1.255–1.699)	<0.001	1.310(1.100–1.560)	0.002
TBil (<17.1μmol/mL vs ≥17.1μmol/mL)	1.034(0.877–1.219)	0.694		
HBsAg (negative vs positive)	1.188(0.966–1.461)	0.102		
Anti-HCV (negative vs positive)	0.856(0.383–1.913)	0.705		
Neutrophil (<3.52 x10^9^/L vs ≥3.52 x10^9^/L)	1.175(1.010–1.366)	0.037	1.130(0.951–1.342)	0.166
Lymphocyte (<1.72x10^9^/L vs ≥1.72 x10^9^/L)	0.866(0.744–1.007)	0.062		
Eosinophils (<0.2x10^9^/L vs ≥0.2 x10^9^/L)	1.154(0.992–1.342)	0.064		
CD3 (<65.90% vs ≥65.90%)	1.030(0.876–1.212)	0.718		
CD4 (<38.80% vs ≥38.80%)	1.176(0.999–1.384)	0.051		
CD8 (<20.15% vs ≥20.15%)	0.949(0.807–1.116)	0.524		
NK (<12.10% vs ≥12.10%)	0.830(0.705–0.977)	0.025	0.820(0.694–0.969)	0.020
B (<13.40% vs ≥13.40%)	1.192(1.013–1.402)	0.035	1.148(0.970–1.358)	0.107
IgG (<12.995g/L vs ≥12.995g/L)	0.973(0.830–1.142)	0.740		
IgM (<1.1g/L vs ≥1.1g/L)	1.078(0.919–1.264)	0.357		
IgA (<2.645g/L vs ≥2.645g/L)	0.992(0.846–1.163)	0.922		
C3 (<0.94g/L vs ≥0.94g/L)	0.913(0.778–1.071)	0.265		
C4 (<0.21g/L vs ≥0.21g/L)	1.026(0.874–1.203)	0.757		

**Abbreviations:** *C*. *sinensis*, *Clonorchis sinensis*; BCLC, Barcelona Clinic Liver Cancer; MVI, microvascular invasion; CEA, carcinoembryonic antigen; AFP, alpha-fetoprotein; CA19-9, carbohydrate antigen 199; HBsAg, hepatitis B surface antigen; Anti-HCV, hepatitis C virus antibody; ALB, albumin; ALT, alanine aminotransferase; AST, aspartate aminotransferase; TBil, total bilirubin; CD3, CD3 T lymphocytes; CD4, CD4 T lymphocytes; CD8, CD8 T lymphocytes; NK, natural killer cells; B, B lymphocytes; IgG, immunoglobulin G antibodies; IgM, immunoglobulin M antibodies; IgA, immunoglobulin A antibodies; C3, complement 3; C4, complement 4.

### *C*. *sinensis* promotes HCC by activating HCC stemness

**Fig 3:**
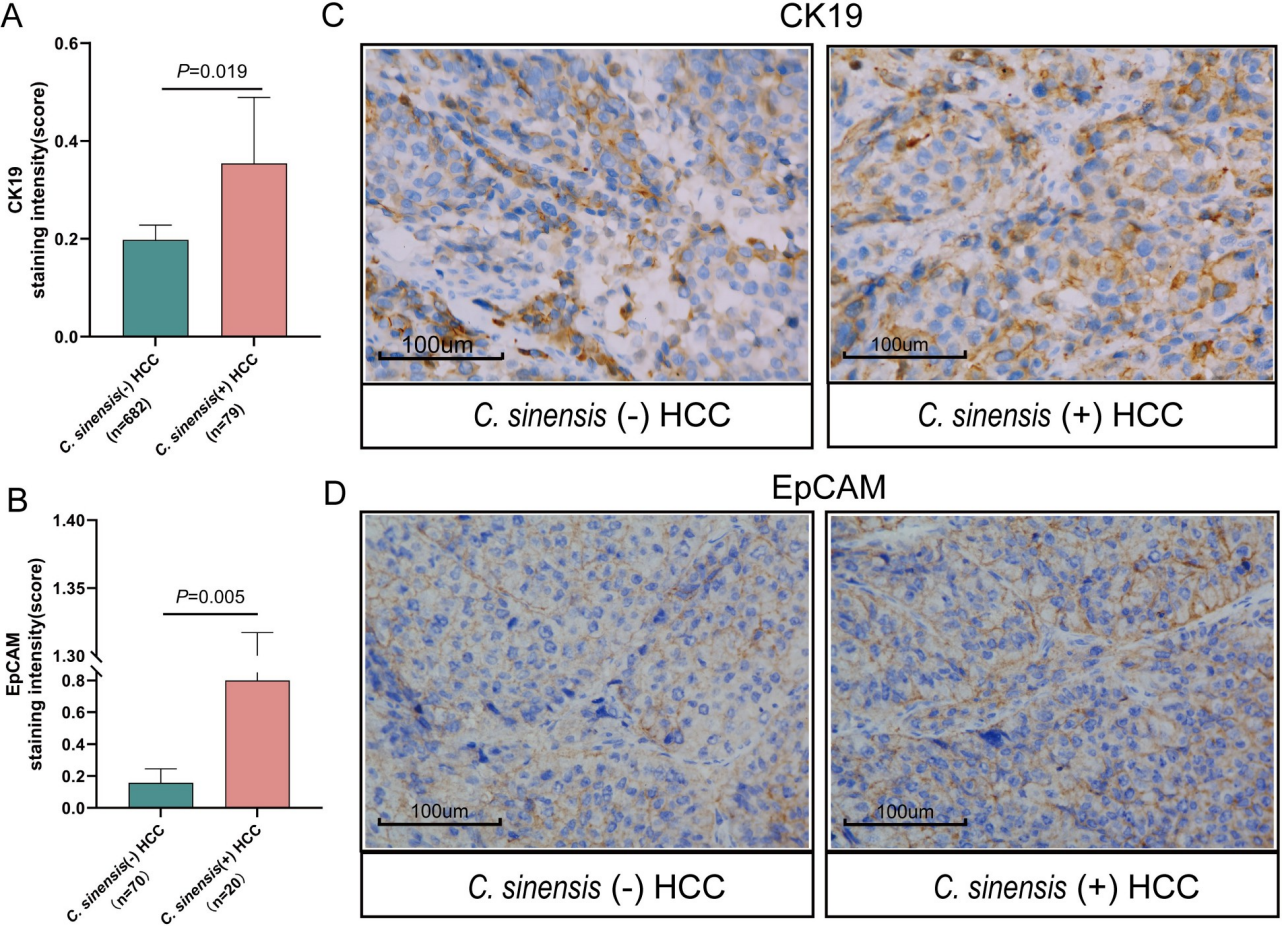
Association between *C*. *sinensis* infection and tumor stem cell markers in HCC patients. (A) CK19 expression, assessed through IHC staining scores, in the *C*. *sinensis* (+) and *C*. *sinensis* (-) HCC groups (Mann–Whitney test, *p* < 0.05); (B) EpCAM expression, evaluated using IHC staining scores, in the *C*. *sinensis* (+) and *C*. *sinensis* (-) HCC groups (Mann–Whitney test, *p* < 0.05); (C) Representative immunohistochemical images of CK19 in HCC tissues of *C*. *sinensis* (+) and *C*. *sinensis* (-) HCC; (C) Representative immunohistochemical images of CK19 in HCC tissues of *C*. *sinensis* (+) and *C*. *sinensis* (-) HCC. **Abbreviations:** *C*. *sinensis*, *Clonorchis sinensis*; HCC, hepatocellular carcinoma; CK19, cytokeratin 19; EpCAM, epithelial cell adhesion molecule.

We hypothesized that *C*. *sinensis* promotes HCC by activating HCC stemness. Positive reactivity for CK19 was observed in 30.38% of *C*. *sinensis* (+) HCC and 20.09% of *C*. *sinensis* (-) HCC lesions. Positive reactivity for EpCAM was observed in 40.00% of *C*. *sinensis* (+) HCC and 15.71% of *C*. *sinensis* (-) HCC lesions. The results showed that the expression levels of CK19 (*p* < 0.05, Fig 3A) and EpCAM (*p* < 0.05, Fig 3B) in liver cancer tissues of *C*. *sinensis*-infected HCC patients were significantly higher than those in noninfected *C*. *sinensis* patients. Fig 3C and 3D are representative images of the expression levels of CK19 and EpCAM in *C*. *sinensis* (-) HCC and *C*. *sinensis* (+) HCC, respectively.

## Discussion

**Fig 4:**
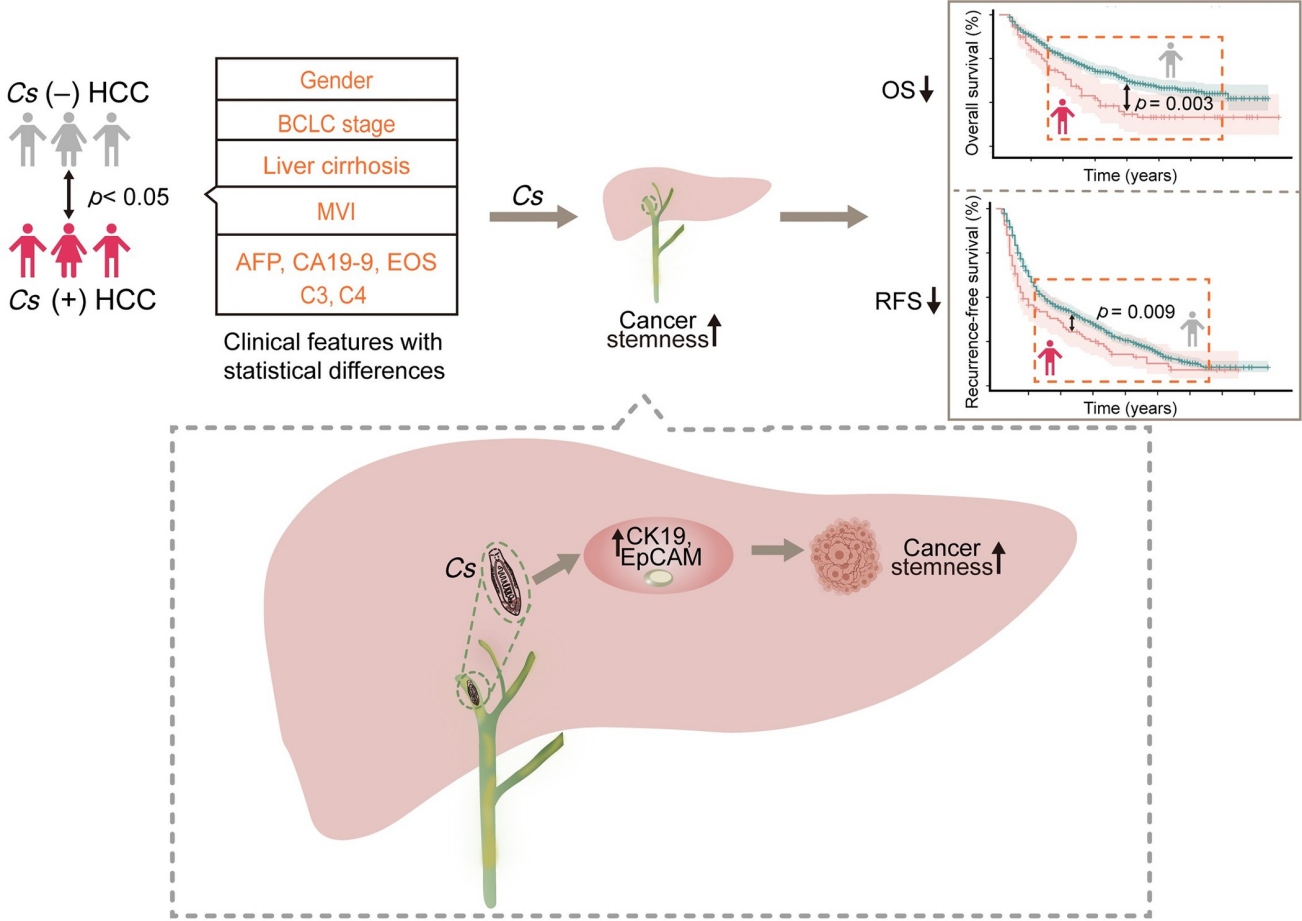
*Clonorchis sinensis* infection predicts unfavorable prognoses by enhancing hepatocellular carcinoma stemness. **Abbreviations:** *Cs*, *Clonorchis sinensis*; HCC, hepatocellular carcinoma; BCLC, Barcelona Clinic Liver Cancer; MVI, microvascular invasion; AFP, alpha-fetoprotein; CA19-9, carbohydrate antigen 199; EOS, Eosinophils; C3, complement 3; C4, complement 4; CK19, cytokeratin 19; EpCAM, epithelial cell adhesion molecule.

Our study presents groundbreaking discoveries regarding the correlations between *C*. *sinensis* infection and HCC. The data demonstrate clear clinicopathological associations between *C*. *sinensis* infection and factors, such as gender, BCLC stage, liver cirrhosis, MVI, AFP, CA19-9, circulating eosinophils and complements. Furthermore, we found that the co-occurrence of *C*. *sinensis* exhibited a significant association with shorter OS and RFS in patients diagnosed with HCC and might be a significant predictive marker for HCC. A major finding was that *C*. *sinensis* has the potential to expedite the malignant progression of HCC through the modulation of cancer stemness. Fig 4 summarizes an overview of how *C*. *sinensis* infection predicts unfavorable prognoses by enhancing hepatocellular carcinoma stemness.

*C*. *sinensis* is a prominent food-borne parasite prevalent in regions such as China, particularly in Guangxi, Guangdong, Heilongjiang and Liaoning [12,29]. This parasite has been associated with various hepatobiliary system injuries, encompassing inflammation, periductal fibrosis, cholangiocarcinoma, and even HCC [20,30]. While a substantial body of evidence links clonorchiasis to cholangiocarcinoma [18,31,32], the connection between clonorchiasis and HCC is less acknowledged. In alignment with Yuan Kuan Li et al.’s research on the correlation between *C*. *sinensis* infection and HCC [9], our results revealed that *C*. *sinensis* was highly related to HCC malignant behavior as exemplified by the BCLC stage and MVI. It was believed that the disparity in *C*. *sinensis* infection rates between sexes primarily stemmed from dietary habits, particularly the consumption of raw fish [33]. This behavior has contributed to a higher prevalence of *C*. *sinensis* infection among males compared to females [9,29]. In the Guangxi Zhuang Autonomous Region, 16.44% of HCC patients were statistically found to be infected with *C*. *sinensis*. Additionally, the prevalence of *C*. *sinensis* in males (14.0%, 95% CI, 13.3–14.8) was significantly higher than in females (7.2%, 95% CI, 6.7–7.8), aligning with the elevated incidence of HCC in males compared to females within the Chinese population. Given these findings, a correlation between the phenomena of *C*. *sinensis* infection and HCC carcinogenesis is evident. However, this correlation does not translate to a strong causation in the absence of experimental validation. Therefore, further experimental validation is necessary to clarify the impact of *C*. *sinensis* infection on HCC carcinogenesis [34–36]. Our data also indicated a higher prevalence of males in the *C*. *sinensis* (+) HCC group than in the *C*. *sinensis* (-) HCC group.

It has been widely recognized that *C*. *sinensis* and other parasitic infections can lead to elevated circulating eosinophils [37]. Moreover, our data also showed circulating eosinophils were more elevated in the *C*. *sinensis* (+) HCC group than in the *C*. *sinensis* (-) HCC group. Eosinophils exert a range of biological effects against helminth parasites, leading to inflammation and damage to affected tissues and even cancers [38,39]. Notable studies have also linked estrogen’s regulatory role to modulating eosinophil values and function [40]. It would be intriguing to explore whether there is a sex-based component to the elevation of eosinophil levels in *C*. *sinensis*-infected HCC. To further explore the underlying mechanisms of eosinophil elevation in *C*. *sinensis*-related HCC, the roles of sex hormones are also worth investigating.

Importantly, with this study, we are the first to report that *C*. *sinensis* (+) HCC patients exhibit elevated circulating complement 3 and 4 levels compared to their *C*. *sinensis* (-) HCC counterparts. Complement 3 and 4 have previously shown high predictive accuracy in distinguishing HCC from controls with chronic HCV infection [41]. Both C3 and C4 have emerged as constituents of tumor-promoting inflammation, implicated in immune modulation by activating protumorigenic neutrophils and tumor-associated macrophages as well as inducing PI3K/AKT-dependent tumor cell proliferation and epithelial-mesenchymal transition [42,43]. However, scant attention has been given to the role of *C*. *sinensis*-mediated Complement 3 and 4 in HCC malignant behavior. Further elucidation is warranted to clarify the impact of *C*. *sinensis* infection on the development of HCC through Complement 3 and 4 mediation.

Cancer stem cells orchestrate a spectrum of crucial roles in hepatocellular carcinoma growth, invasion, metastasis, recurrence, and therapy resistance [44]. *C*. *sinensis*, a carcinogenic human liver fluke, fosters chronic inflammation, epithelial hyperplasia, periductal fibrosis, and even carcinogenesis through prolonged infection [17]. Recent research highlights various mechanisms through which *C*. *sinensis* contributes to HCC progression. The excretory/secretory products of *C*. *sinensi*s inhibit HCC cell apoptosis via mitochondria-mediated pathways triggered by Ca^2+^ disruptions [11,30]. Additionally, *C*. *sinensis* granule protein promotes liver cell malignant transformation, migration, and invasion by activating the ERK and PI3K/AKT pathways [10]. However, the role of *C*. *sinensis* in promoting human HCC development via cancer stemness regulation remains less understood.

Our findings underscore that *C*. *sinensis* not only serves as an independent risk factor for OS and RFS but also elevates the expression of HCC CSC markers, namely, CK19 and EpCAM. CK19+ HCC is an aggressive subtype characterized by its propensity for early recurrence, metastasis and chemotherapy tolerance as well as poor prognosis [45–48]. Oriana Miltiadous et al. reported an independent association between CK19 and HCC recurrence, with a hazard ratio of 2.95 [49]. Meanwhile, the EpCAM+ HCC subtype exhibited characteristics of hepatic stem cells, displaying the capacities for self-renewal and differentiation, as well as the capability to initiate highly invasive HCC [50–52]. This leads us to propose an additional mechanism by which *C*. *sinensis* infection promotes HCC progression that involves the enhancement of cancer stemness in tandem with the above three mechanisms.

Mounting evidence indicates that *C*. *sinensis* infection drives Th2 immune responses and facilitates the production of the cytokines IL-13, TGF-β, IL-10 and IL-4 [53,54], which have been implicated in promoting cancer stemness [55,56]. Carlo De Salvo et al. uncovered the importance of eosinophils in the early cascade leading to intestinalized metaplasia in gastritis-prone mice, suggesting a potential mechanism that contributes to the inflammation-metaplasia-dysplasia-carcinoma sequence [57]. Our observations revealed higher eosinophil counts in *C*. *sinensis* (+) HCC patients than in *C*. *sinensis* (-) HCC patients. The underlying mechanism through which *C*. *sinensis* infection drives eosinophils or Th2 immune responses affecting HCC stemness needs to be fully investigated.

While our study has yielded promising outcomes, there are still some limitations to be considered. First, the sample size, particularly within the cohort of *C*. *sinensis*-related HCC patients, is relatively modest. To validate and reinforce our findings, a more extensive sample size is essential. Second, our investigation primarily centered around the correlation between *C*. *sinensis* infection and circulating immune cells, neglecting potential alterations within the HCC tumor microenvironment. Expanding our focus to include these changes could provide a more comprehensive understanding. Third, although our study has illuminated that *C*. *sinensis* contributes to heightened HCC stemness, the precise underlying mechanism behind this phenomenon remains unexplored. Investigating this mechanism is imperative to fully grasping the intricacies of our findings.

Our research uncovers a hitherto overlooked association between *C*. *sinensis* infection and HCC, offering crucial insights into both clinical implications and underlying mechanisms. This study underscores the importance of incorporating *C*. *sinensis* infection into the assessment and management of HCC patients, particularly in regions where this parasitic infection is endemic. Given the observed diminished prognosis among HCC patients with *C*. *sinensis* infection following hepatectomy, treatment strategies may necessitate targeted interventions tailored to address this specific subgroup. Public health policies may benefit from the integration of systematic screening for *C*. *sinensis* in populations at heightened risk by enhancement of early detection and intervention. Future research endeavors should delve into the molecular mechanisms through which *C*. *sinensis* infection influences the initiation and progression of HCC. A comprehensive understanding of these pathways holds the potential to inform targeted treatment and intervention strategies.

In conclusion, our study provides robust evidence that *C*. *sinensis* infection is strongly linked to unfavorable prognoses in HCC patients due to its capacity to enhance HCC stemness. These results contribute to a more comprehensive comprehension of the interplay between *C*. *sinensis* and HCC, shedding fresh light on the carcinogenic potential of *C*. *sinensis*. To solidify these findings, further endeavors should encompass large multicenter randomized controlled studies and comprehensive investigations into the intricate mechanisms at play.

## Supporting information

S1 Data The raw data of this study. (XLSX)
